# Fidelity monitoring across the seven studies in the Consortium of Hospitals Advancing Research on Tobacco (CHART)

**DOI:** 10.1186/s12971-015-0056-5

**Published:** 2015-09-03

**Authors:** Sonia A. Duffy, Sharon E. Cummins, Jeffrey L. Fellows, Kathleen F. Harrington, Carrie Kirby, Erin Rogers, Taneisha S. Scheuermann, Hilary A. Tindle, Andrea H. Waltje, the Consortium of Hospitals Advancing Research on Tobacco (CHART)

**Affiliations:** Ohio State University, College of Nursing, Newton Hall, 1585 Neil Ave, Columbus, OH 43210 USA; VA Center for Clinical Management Research, HSR&D Center of Excellence, 2215 Fuller Road, Ann Arbor, MI 48105 USA; Department of Family Medicine and Public Health, University of California, San Diego, 9500 Gilman Drive, MC0905, La Jolla, CA 92093 USA; Kaiser Permanente Center for Health Research, 3800 N Interstate Ave, Portland, OR 97227 USA; Division of Pulmonary Medicine, University of Alabama at Birmingham, 1900 University Blvd., THT541-G1, Birmingham, AL 35294-0006 USA; Moores Cancer Center, University of California, San Diego, 9500 Gilman Drive, MC0905, La Jolla, CA 92093 USA; Department of Population Health, New York University School of Medicine, 227 E. 30th St., New York, NY & VA New York Harbor Healthcare System, 423 E. 23rd St., New York, NY USA; Department of Preventive Medicine and Public Health, University of Kansas Medical Center, 3901 Rainbow Blvd, Kansas City, KS 66160 USA; Department of Medicine, Vanderbilt University School of Medicine, 2525 West End Avenue, Suite 370, Nashville, TN 37203 USA; University of Michigan, Internal Medicine, Brehm Tower, Room 6115, 1000 Wall Street, Ann Arbor, MI 48109-5714 USA

**Keywords:** Smoking, Cessation, Inpatient, Fidelity

## Abstract

**Background:**

This paper describes fidelity monitoring (treatment *differentiation, training, delivery, receipt and enactment*) across the seven National Institutes of Health-supported Consortium of Hospitals Advancing Research on Tobacco (CHART) studies. The objectives of the study were to describe approaches to monitoring fidelity including treatment differentiation (lack of crossover), provider training, provider delivery of treatment, patient receipt of treatment, and patient enactment (behavior) and provide examples of application of these principles.

**Methods:**

Conducted between 2010 and 2014 and collectively enrolling over 9500 inpatient cigarette smokers, the CHART studies tested different smoking cessation interventions (counseling, medications, and follow-up calls) shown to be efficacious in Cochrane Collaborative Reviews. The CHART studies compared their unique treatment arm(s) to usual care, used common core measures at baseline and 6-month follow-up, but varied in their approaches to monitoring the fidelity with which the interventions were implemented.

**Results:**

Treatment *differentiation* strategies included the use of a quasi-experimental design and monitoring of both the intervention and control group. Almost all of the studies had extensive *training* for personnel and used a checklist to monitor the intervention components, but the items on these checklists varied widely and were based on unique aspects of the interventions, US Public Health Service and Joint Commission smoking cessation standards, or counselor rapport. *Delivery* of medications ranged from 31 to 100 % across the studies, with higher levels from studies that gave away free medications and lower levels from studies that sought to obtain prescriptions for the patient in real world systems. Treatment *delivery* was highest among those studies that used automated (interactive voice response and website) systems, but this did not automatically translate into treatment *receipt* and *enactment*. Some studies measured treatment *enactment* in two ways (e.g., counselor or automated system report versus patient report) showing concurrence or discordance between the two measures.

**Conclusion:**

While fidelity monitoring can be challenging especially in dissemination trials, the seven CHART studies used a variety of methods to enhance fidelity with consideration for feasibility and sustainability.

**Trial registration:**

- Dissemination of Tobacco Tactics for hospitalized smokers. Clinical Trials Registration No. NCT01309217.
- Smoking cessation in hospitalized smokers. Clinical Trials Registration No. NCT01289275.
- Using “warm handoffs” to link hospitalized smokers with tobacco treatment after discharge: study protocol of a randomized controlled trial. Clinical Trials Registration No. NCT01305928.
- Web-based smoking cessation intervention that transitions from inpatient to outpatient. Clinical Trials Registration No. NCT01277250.
- Effectiveness of smoking-cessation interventions for urban hospital patients. Clinical Trials Registration No. NCT01363245.
- Comparative effectiveness of post-discharge interventions for hospitalized smokers. Clinical Trials Registration No. NCT01177176.
- Health and economic effects from linking bedside and outpatient tobacco cessation services for hospitalized smokers in two large hospitals. Clinical Trials Registration No. NCT01236079.

## Introduction

Treatment fidelity is the extent to which an intervention is implemented as designed [1]. The rationale for monitoring fidelity is to address the methodological strategies used to monitor and enhance reliability and validity of the intervention [2]. Moreover, fidelity speaks to the degree of integrity of an intervention. Ensuring treatment fidelity gives researchers more confidence in their results [2] and increases credibility [3], particularly in trials conducted in community settings where there are diverse settings, therapists, and patients [4, 5].

Lack of fidelity refers to any gap between the intervention as it was planned and the intervention as it was actually implemented [6]. If a treatment was not given completely as designed and statistical effects of the treatment are non-significant, then the treatment may be discarded prematurely. Indeed, higher treatment fidelity has been linked to improved outcomes [6]. If significant effects were found, but fidelity was not monitored, then it is unclear whether the difference was due to the intervention itself or to something else [2, 7]. Fidelity measures can be monitored and included in statistical analysis (e.g., the number of times an individual subject was reached on the phone to assess the implementation of a phone intervention) and therefore increase knowledge about how the components of an intervention influence the outcome. Finally, the assessment of treatment fidelity serves to identify barriers to implementation that need to be addressed and modified in order to ensure that the intervention has a greater chance of sustainability [8].

The five main components of fidelity monitoring are well established and include study *design, training, delivery, receipt, and enactment* [2, 9, 10]. Study *design* monitoring includes consideration on whether or not the intervention is sufficiently *differentiated* from the control group (avoidance of cross-contamination). Standardized *trainings* of providers/interventionists support standardized treatment delivery. In addition, monitoring and maintaining provider skills over time is a necessity. Treatment *delivery* measures whether or not the treatment was delivered as intended. Treatment *receipt* measures whether or not the participant understands the intervention and indicates confidence and ability to apply the skills learned in the intervention. Treatment *enactment* measures whether or not the participant participated in the intervention.

While many studies have been published on the importance of fidelity monitoring and some have published results from their own studies, no papers that we know of have published approaches to fidelity monitoring across several smoking cessation studies. Hence, this paper will describe the methods of fidelity monitoring and provide fidelity data across seven studies that were funded by the National Institutes of Health (NIH) to disseminate unique smoking cessation interventions to hospitalized smokers. Since fidelity influences both the degree to which changes can be attributed to the intervention (internal validity), and the ability to replicate and disseminate the intervention (external validity) [11], the assessment of treatment fidelity across the seven studies may be useful to researchers conducting smoking cessation and other behavioral intervention trials [7, 9–13].

## Methods

Collectively called the Consortium of Hospitals Advancing Research on Tobacco (CHART), these studies conducted between 2010 and 2014 recruited over 9500 inpatient cigarette smokers to test the effectiveness of these unique smoking cessation interventions. The protocols of these seven studies have been published [14–21] and outcome papers will follow. All of the CHART studies implemented the components of smoking cessation interventions shown to be efficacious in Cochrane Collaborative Reviews [22] including medication and behavioral counseling and follow-up calls. Although usual care varied across the seven studies, all of the CHART studies compared their unique treatment arm(s) to usual care and used common core measures at the baseline and 6-month follow-up. However, each study was unique in how fidelity was monitored. A brief overview of each study design follows.

### University of Michigan Medical Center (UMMC)

The Tobacco Tactics study used a quasi-experimental design in five Michigan Trinity Health System Hospitals. Nurses in three experimental hospitals were taught to conduct the in-hospital, face-to-face Tobacco Tactics intervention in a 1-h educational session using the Tobacco Tactics toolkit. Nurses in the other two hospitals continued usual care (brief advice and brochure). Nurses and inpatient smokers (*N* = 1528) regardless of motivation to quit in both the experimental and control sites were surveyed pre- and post-implementation of the intervention [15].

### University of California, San Diego (UCSD)

In this 2 by 2 factorial randomized controlled trial (RCT), 1270 inpatient smokers motivated to quit from three healthcare systems (a total of 5 hospitals) in San Diego and Davis, CA were randomized to one of four conditions: 1) usual care; 2) nicotine patches provided at the time of discharge; 3) telephone counseling after discharge and; 4) nicotine patches at discharge plus telephone counseling after discharge. Six standard counseling sessions were proactively provided by the state quitline post discharge. Subjects in the patch conditions received 8 weeks of nicotine patches at the time of discharge [16].

### University of Kansas (KU)

This RCT compared warm-handoff versus fax referral (usual care) for linking hospitalized smokers motivated to quit to state quitline services (*N* = 1054) from two large hospitals. Counselors provided brief cessation advice to all patients and provided them with a standard smoking cessation booklet. In the warm hand-off intervention condition, patients received an abbreviated bedside intervention and the counselor called the quit line and transferred the call to the patient’s bedside hospital phone or mobile phone before leaving. In the control condition, fax-referral patients received the usual care cessation counseling and were fax referred to the quitline. Outcome data collected from the state quit line provider, Alere Well Being (AWB), included enrollment and number of calls completed out of a total of 5 intervention calls [21].

### New York University (NYU)

This RCT compared two methods of smoking cessation delivered to all inpatient smokers regardless of motivation to quit discharged from two urban public hospitals in New York City (Bellevue and the Manhattan VA): 1) seven sessions of proactive telephone counseling delivered by study staff; or 2) referral to the New York State Quitline via fax or online referral (*n* = 805). Counselors in the intervention arm assisted patients (*n* = 805) in obtaining four weeks of NRT patch or gum after discharge. Counselors in the Quitline arm assisted patients (*n* = 814) in obtaining NRT following usual Quitline protocols (*N* = 1619). For most participants this was two weeks of NRT patch or gum [17].

### University of Alabama at Birmingham (UAB)

This RCT randomized all smokers regardless of motivation to quit in a large university hospital who had an email address and access to the internet to: 1) usual care; or 2) the web-based Decide2Quit intervention. The intervention included a visit by a counselor who introduced them to the web-site, assisted them with registering to the site, and provided a booklet that gave an overview of the web-site, including information to help them log into the site on their own (*N* = 1488). If the participant was discharged prior to the bedside registration, a booklet was sent to their home address and a counselor walked them through website registration and orientation over the phone. Questions completed during the intervention registration informed tailored email messages automatically sent on a scheduled basis. The website allowed participants to send messages to a counselor and receive responses. Participants were called between 10 and 30 days post hospitalization to encourage use of the web-site [18].

### Kaiser Permanente Center for Health Research (KPCHR)

The Inpatient Technology-Supported Assisted Referral (ITSAR) study was a RCT recruiting smokers motivated to quit in three large hospitals serving the Portland OR metropolitan area that compared: 1) adding an assisted referral (AR) to available outpatient quit services and medications following discharge and four post-discharge Interactive Voice Response (IVR) telephone follow-up calls (AR + IVR); and 2) usual care bedside cessation counseling, medication, and information about available quit services. IVR calls captured smoking status, cessation program enrollment status, medication use, and provided brief supportive messages. Nine hundred participants were recruited (599 to AR + IVR and 301 to usual care) using a 2:1 recruitment strategy [19].

### Massachusetts General Hospital (MGH)

The RCT Hospital-Initiated Assistance for Nicotine Dependence 2 (Helping HAND 2) is a multisite iteration of Helping HAND 1 [23] and is enrolling 1350 adult smokers motivated to quit admitted to 3 acute care hospitals in Massachusetts and Pennsylvania. Data are included on 529 participants who were enrolled at the time of this writing. All subjects received brief in-hospital smoking intervention and were randomly assigned at discharge to either usual care (referral to the Massachusetts or Pennsylvania state quitline) or extended care intervention, which consisted of a 3-month program with 2 components: 1) free medication (30-day supply of FDA-approved medication including nicotine replacement, bupropion, or varenicline) given at hospital discharge and refillable free for a total of 90 days to facilitate medication use and adherence; and 2) TelASK (participating IVR company) triage to telephone counseling from a national quitline provider, Alere Well Being (AWI) [20].

## Results

**Fig. 1:**
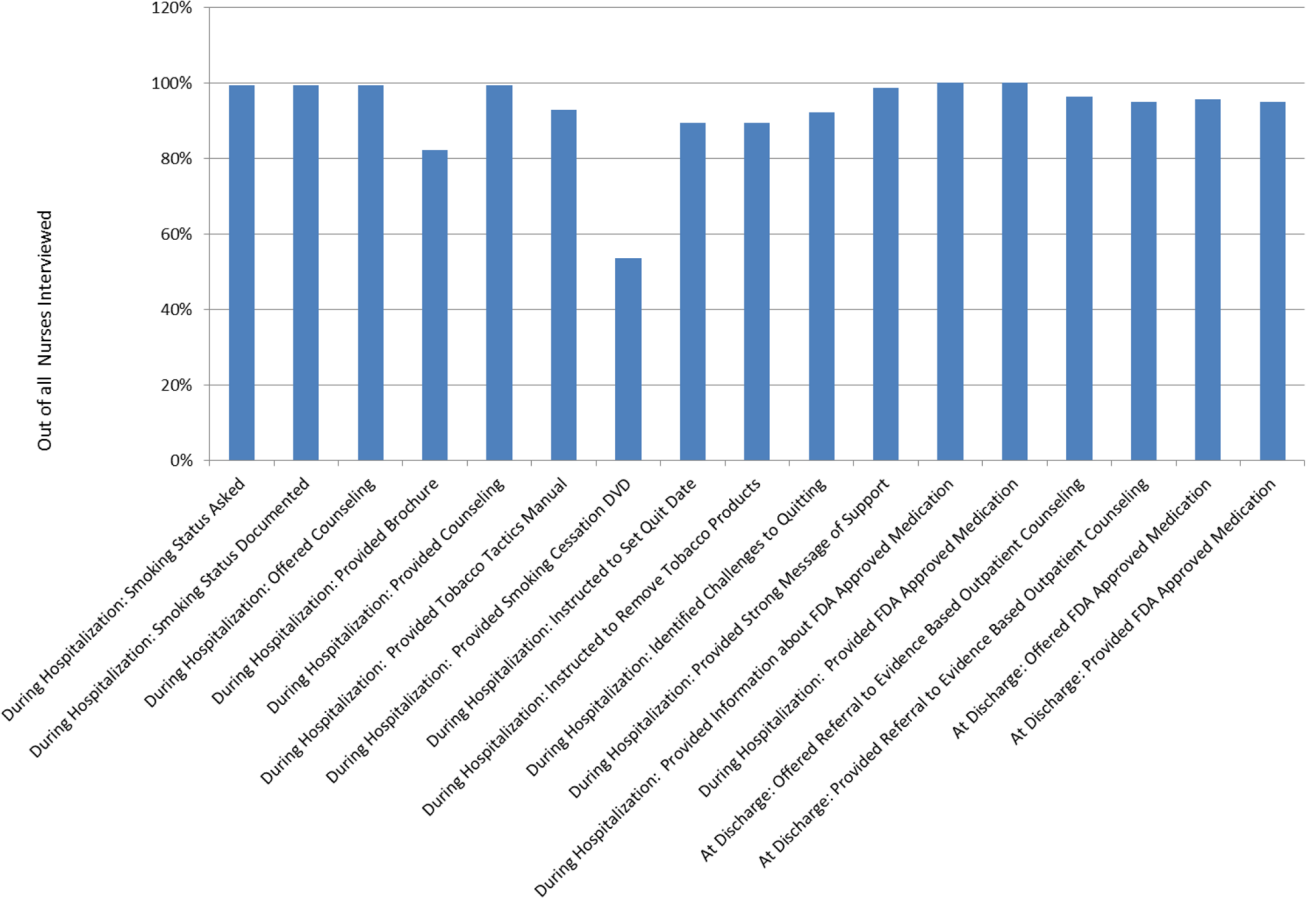
(UMMC): Self-Reported Treatment *Delivery* by Nurses Interviewed (*n* = 140)

Table 1 summarizes how each of the five components of fidelity were addressed and monitored by each of the seven CHART studies. Selected results of the fidelity data from each site follows. Figure 1 and Tables 2, 3, 4, 5, 6 and 7 show examples of the components of fidelity monitoring from each site with corresponding data.

**Table 1:** Components of fidelity addressed by each study

	Study design/Differentiation	Training	Delivery	Receipt	Enactment
UMMC	Quasi-experimental design with 3 intervention hospitals and 2 usual care hospitals decreased chances of cross-over.	Packaged both nurse training and patient intervention into a toolkit.	Pre- post-intervention nurse surveys in intervention and control sites.	30 day-post-intervention patient surveys.	30-day post-intervention patient surveys.
	Increased chances of sustainability as all nurses in intervention sites were trained.	Research nurse trained trainers until they demonstrated fidelity of training.	Nurse interviews in intervention sites only.	EMR download medications and counseling.	
		Patient intervention manualized.	Also see Fig. 1.		
			EMR download from nurse documentation.		
UCSD	Randomization was completed using iPads and tablets so recruiters had timely access to randomized condition, minimizing data entry errors and cross-contamination of intervention.	An operations manual guided research staff on the various components of the study.	Reports were generated from the comprehensive UCSD database (that combined both research-specific data with intervention data) to ensure protocol adherence (e.g., proper number of attempts for counseling clients, nicotine patches distributed to clients, materials mailed, etc.).	Treatment receipt was assessed by monitoring of quitline counseling database, hospital documentation of patch delivery, and self-report at follow-up regarding receipt and use of nicotine patches and/or quitline services or other tobacco treatment.	Counseling adherence data was collected from the quitline documenting the number of calls participants completed.
		Standardized training that included role-play, was provided to recruitment staff (i.e., respiratory therapists and dedicated research recruiters) and to quitline staff responsible for providing the counseling intervention.	Also see Table 2.		Two- and six-month evaluation Calls contained self-reported use of quitting aids (including those from the study) and use of behavioral treatment (including quitline).
		The project manager went into the field quarterly, or when new employees were hired, observed, and provided feedback to staff to ensure adherence to project protocols.	The counseling used a structured protocol.		
		Bi-weekly meetings allowed quitline counselors to discuss specific counseling cases and review skills, increasing fidelity.	Timing, length, and frequency of counseling calls was recorded.		
KU	Quitline Alere Well Being (AWB) provided reports on how the patient had been referred to the quitline—fax or warm handoff.	A master’s degree level certified tobacco treatment specialist trained hospital tobacco use counselors.	Trainers observed counselors’ delivery of counseling and counselor’s documentation of the intervention.	Treatment receipt was assessed by hospital treatment counselor documentation and self-report at follow-up as to whether participants received quit line services or other tobacco treatment.	Counseling adherence data was collected from the quitline documenting the number of calls participants completed.
	Differentiation was assessed by comparing records of group assignment to the group file in which AWB reported data back to the research team.	Hospital counseling training included didactics, role-playing, and supervised delivery of the intervention.	Fidelity monitors observed a 10 % convenience sample of the tobacco use treatment sessions delivered in the hospital.		
		During the training process, the fidelity monitor observed new trainee counselors at least once per week.	A checklist was used to assess provision of each component of treatment by intervention arm for the study.		
		Hospital tobacco treatment counselors used a counseling checklist to document assessment, smoking cessation medication usage and recommendations, and referrals to the quitline.	Also see Table 3.		
			Fidelity monitors assessed how well counselors documented in the medical record the treatment that was provided.		
NYU	The processes of transferring participant data to the Quitline (control) and providing multi-session telephone counseling (intervention) were accomplished by different study team members, limiting chance of cross-over.	Standardized operating procedure (SOP) manuals were developed for all study procedures.	Intervention counselors completed standardized documentation of their counseling sessions using a study database with close-ended and open-ended fields to document the number and duration of counseling calls, correctness of contact information, overall success in reaching participants, NRT orders, and topics covered.	Treatment receipt (i.e., patient understanding of the intervention and confidence) was not systematically assessed.	Enactment was assessed by counselor documentation and 2-month patient follow-up surveys.
	Differentiation was assessed by comparing participant group assignments with Quitline and intervention counselor documentation.	Intervention counselors underwent 20–30 h of initial training on the counseling protocol.	Also see Table 4.	2-month follow-up surveys assessed patient satisfaction with treatment.	Also see Table 4.
		Training included didactic lectures, role-plays and practice with standardized patients (actors trained to portray real patients).	Each month a random sample of intervention counseling sessions were audiotaped and reviewed by the study’s clinical supervisor using a standardized form assessing adherence to the protocol and counseling approach.		
		Intervention counselors participated in weekly supervision with the study’s clinical supervisor providing an opportunity for training updates (e.g., review of the protocol and counseling approaches).	The study supervisor met with counselors individually to review the form and provide feedback.		
UAB	Treatment differentiation occurred by limiting access of the web-site to those randomized to the intervention through registration to the website by an intervention staff member.	Hospital staff attended at least one two-hour training session.	Once registered, participants received automated emails.	The website tracked messages sent to and from the Tobacco Treatment Counselor.	The website tracked participants’ web-site log-ins, number of days website accessed, and number of web pages visited.
			The website tracked registrations, log-ins and automated email messages and subset of participants were surveyed.	Also see Table 5.	Also see Table 5.
KPCHR	Counselor documented assisted referral acceptance, completion of referrals to outpatient counseling, and documented discharge medication orders.	Study staff were trained and certified in Good Clinical Practice.	Counselor completed a tobacco consult checklist for each smoker seen, and a study enrollment checklist for consented and randomized patients.	Counselor documented consult topics discussed with the patient, assisted referral acceptance and referrals, and discharge medication orders.	Utilization of quit resources was documented at 6-month follow-up and electronic medical records where available.
	Counselor documented receipt of printed quit information for patients in the control group.	Counselors attended the same training sessions on tobacco-dependence treatment delivery for hospitalized patients.	Counselors were monitored during initial piloting, and participated in ongoing case management discussions among the counselor group.	IVR call attempts, call completions, and responses, were monitored electronically.	
	Documentation, including IVR completion data, was tracked and reviewed monthly by study staff.	Other staff received appropriate training in use of the study’s electronic data management system at each site and in coding rules to complete the forms properly.		Also see Table 6.	
	A participant screening and tracking protocol was used to identify patients with subsequent hospital admissions to prevent reenrollment.				
MGH	Medication was provided exclusively to intervention participants at the time of hospital discharge (verified by study ID).	For smoking cessation medication, study staff were trained by the overall coordinator and site coordinators to obtain medication from the inpatient pharmacy and deliver it to intervention participants’ bedside at the time of hospital discharge.	Medication – Study staff delivered 1 month of smoking cessation medication to the patient’s bedside.		Study staff electronically tracked medication dispensation via a standard database.
	Similarly, only intervention participants were entered into the IVR database to receive calls after hospital discharge (determined by study ID).	Staff were also trained to enter intervention participants’ information into the IVR database so that they could be called according to the TelASK (participating IVR company) protocol).	IVR calls were initiated by TelASK with automated telephone calls to smokers.	The study did not formally track treatment receipt.	IVR calls were monitored by TelASK and study staff via secure access to a web-portal. Telephonic behavioral counseling enrollment and calls were monitored by AWI via standard database.
			All call activity was recorded electronically via call flow sheets, and a subset of calls was randomly selected by TelASK for internal quality review.		
			Also see Table 7.

**Table 2:** (UCSD): *delivery* of nicotine patches by condition (*N* = 637)

	Total patch conditions (n = 637)	Patch only (n = 320)	Counseling + patch (n = 317)
	%	%	%
Received patches at discharge	62.8	67.5	58.0
Received patches by mail	35.5	31.3	39.8
Refused patches	1.7	1.2	2.2

**Table 3:** (KU): hospital counselor treatment *delivery* as measured by checklist and direct observation (*N* = 108)

Warm handoff (*N* = 57 observations)	% performed appropriately
Let patient know survey is over and now you will be moving into the treatment portion of the intervention.	98
Describe warm hand-off process	79
Explain to patient you will now call the quit line then hand them the phone	100
Explain they will talk to two people: first registration and second they will be transferred to the quit coach	98
Explain the registration person will ask some questions that might seem redundant (like we have already asked)	79
Let them know you will check back in on them after the call	100
Perform call, using appropriate language	100
Leave room, notify patient’s nurse or floor staff patient is talking to quit line, discuss medications for withdrawal if appropriate.	97
Fax Referral (*N* = 51 observations)	
Let patient know survey is over and now you will be moving into the counseling/ treatment portion of the intervention	98
Conduct assessment of smoking history readiness to quit	96
Briefly touch on benefits and barriers to quitting as appropriate	100
Provide accurate medication information, use elicit, provide, elicit	94
Build plan to quit/stay quit using the booklet (as needed)	94
Ask if patient requests cessation medication script on discharge	98
Summarize key topics, goals, and next steps	94
Description of Fax Referral Process	
Someone will call at the number you provided soon after discharge	96
Explain they will talk to registration first and answer questions before talking to a counselor	75
Avoid telling patient they don’t have to take all calls	98
Discuss plans to obtain medications post discharge: 1. Discuss patient interest in post discharge medications. 2. Tell them we will request a prescription from medical staff if appropriate. 3. Discuss how to obtain medication	96
Follow up with medical staff about inpatient and discharge medications	90

**Table 4:** (NYU): *treatment delivery* and *enactment* intervention arm from counselor documentation and patient surveys (*N* = 805)

	% performed appropriately
Medications from Counselor Documentation	
Interested in receiving Nicotine Replacement Therapy (NRT) NRT from the study^a^	32
NRT provided by the study	31
Received a NRT prescription at discharge	8
Calls from Counselor Documentation	
Completed at least one call	52
Completed 7 calls	14
Set a Quit Date	10
Used NRT	27
Two-month Patient Survey	
Used telephone counseling	48
Used NRT	34

^a^Data only available on participants who spoke with an intervention counselor

**Table 5:** (UAB): treatment *receipt* and *enactment* from web-system (*n* = 748) and a sub-set of participant self-reports at 6 months post-intervention (*n* = 172)

Website logins^a^	% system reported *n* = 748	% participant reported *n* = 172
Never	6	32
1–2 times	78	28
3–5 times	11	24
6–10 times	3	10
More than 10 times	2	6
Intervention emails	% system reported sent	% participant reported received
0	1	12
1–3	0	13
4–10	0	32
More than 10	99	43
Counselor messages via web-site	% System reported messages to counsel or (range 0–17)	
0	38	
1	51	
2	6	
3 or more	5	

^a^One login was during registration

**Table 6:** (KPCHR): tobacco cessation *treatment receipt* among ITSAR intervention group recipients (AR + IVR) obtained from consult and enrollment checklist and IVR service provider reports (*N* = 597)

	All sites *N* = 597	Kaiser Sunnyside Medical Center *N* = 414	Legacy Emanuel/ OHSU Hospitals *N* = 183
	%	%	%
Inpatient quit services			
Withdrawal/comfort assessed	99	99	100
Tobacco use/quit history	98	98	100
Contraindications for meds	95	93	100
Risk factors for quitting	95	94	100
Discussed quit services	99	99	100
Provided quit materials	99	99	100
Assisted referral to outpatient care			
HES/fax to quitline	57	49	78
D/C meds arranged	43	50	27
Both	28	30	23
Primary care physician notified of quit program enrollment	76	98	25
IVR completed calls (post D/C)			
Call 1	53	52	56
Call 2	48	51	41
Call 3	41	41	41
Call 4	34	37	28

**Table 7:** (MGH): *treatment delivery* (Medication, IVR Calls) and *treatment enactment* (Enrollment in Behavioral Telephone Counseling) among *Helping HAND 2* trial participants (*N* = 529)

Components of enhanced care	Percent
Pharmacotherapy	
Received First Month Pharmacotherapy on Hospital Discharge	100
NRT Monotherapy	22
NRT Combination Therapy	74
Bupropion	<1
Bupropion + NRT	<1
Varenicline	<1
Varenicline + NRT	<1
Received 1st refill	51
Received 2nd refill	19
Interactive Voice Response (IVR)	
# IVR Calls Accepted	
0	12
1	12
2	16
3	21
4	16
5	24
Number of calls accepted among those who enrolled in behavioral counseling via warm transfer (*n* = 134)	
1	40
2	20
3	16
4	10
5	13

### University of Michigan Medical Center (UMMC)

Figure 1 shows the results for treatment *delivery* assessed using the nurse interviews conducted with 11 % (*n* = 140) of nurses in the intervention sites. Nurses reported high *delivery* rates on most items, except for showing the DVD. Qualitative comments from the nurses indicated that the overhead television system was often not working or when it was working, it was cumbersome to use. One nurse who did not attend the training reported delivering the intervention as she learned it from the other nurses.

### University of California, San Diego (UCSD)

A chart audit showed that treatment *differentiation* was high in that no patients in the control or counseling only group were given nicotine patches at discharge. Examination of the quitline data revealed that 10 subjects (<1 %) in the control or patch only group proactively called the quitline after hospital discharge and received counseling. Table 2 shows an example of monitoring the treatment *delivery* comparing the rates for those in the patch only condition to those in the counseling plus patch condition. Overall 55.1 % received their patches upon discharge, 34.5 % were mailed patches after discharge, 1.7 % refused, and 8.6 % were unknown. There was greater difficulty delivering patches to subjects in the combined counseling plus patch condition than the patches only condition (58.0 % versus 67.5 %; p < .05).

### University of Kansas (KU)

Table 3 shows fidelity data for treatment *delivery* assessed using 108 direct observations of counselors in both the intervention (*n* = 57) and control (*n* = 51) arms. The mean fidelity was 94 % for both the treatment and control arms. In terms of treatment *enactment*, 99.6 % of participants assigned to warm hand-off intervention enrolled, while only 59.6 % of participants assigned to fax referral control arm enrolled (p < .001). The rates of completed calls were as follows: 31 % did not complete any calls, 25 % completed 1 call, 16 % completed 2 calls, 12 % completed 3 calls, 9 % completed 4 calls, and 7 % completed 5 calls.

### New York University (NYU)

Table 4 shows treatment *delivery* for smoking cessation medications; 32 % of intervention participants were interested in receiving NRT, 31 % were delivered NRT, and 8 % received a NRT prescription on hospital discharge. In terms of treatment *enactment*, 52 % of participants randomized to the intervention arm (*n* = 805) completed at least one counseling call and only 14 % received all seven counseling calls. Participants completed an average of two counseling calls (the average was four calls among participants who began counseling). The average length of the first call was 22 min and follow-up calls averaged 13 min. Eighty-one participants set a quit date with their counselor (10 % of all intervention participants; 20 % of those who began counseling) and 218 participants reported using NRT to a counselor (27 % of all intervention participants; 52 % of those who began counseling). Similarly, 2-month follow-up surveys showed that 48 % of intervention participants (384/805) reported receiving telephone counseling and 34 % (271/805) reported using NRT.

### University of Alabama at Birmingham (UAB)

Treatment *enactment* was monitored using web-system tracking of logins, the number of emails automatically sent, and the number of emails sent to and responded by the tobacco counselors as well as by patient recall of web participation from a subgroup of participants (see Table 5). Of the 748 assigned to the web intervention, 735 (98 %) participants were registered, 73 % at bedside and 25 % over the telephone. Post-hospitalization calls by interventionists encouraging use of the website were completed for 64 % of intervention participants, while another 34 % were left detailed messages encouraging use. The web-system documented at least 1 web-page was visited by 700 (94 %) participants with the mean number of pages accessed being 5.4 (sd = 4.1; range 1–29). Ninety-one participants (11 %) emailed the tobacco counselor two or more times, while another 51 % emailed the counselor at least once. When a subset of participants (22 %; *n* = 167) were asked how often they read their intervention emails, 27 % of respondents reported always, 19 % often, 24 % half the time, 13 % rarely, 5 % never and 12 % did not respond. Participants were also asked for comments regarding their experience with the intervention, providing some insight into why the website wasn’t used as much as planned (e.g., computer problems, not comfortable with using a website, found it confusing) and how the emails were perceived (e.g., boring, interesting, helpful, incessant).

### Kaiser Permanente Center for Health Research (KPCHR)

Treatment *delivery* was assessed using a treatment form to record components of counselor-patient discussion of tobacco use variables and an enrollment checklist to document assisted referral (AR) to outpatient counseling and medications. Among the 597 study participants who were randomized to the AR + IVR group, nearly all participants received nicotine withdrawal counseling, discussed tobacco use and quit history, were assessed for medication contraindications, discussed available quit services, and were provided printed quit materials (Table 6). Patients randomized to the AR + IVR group were offered a referral to centralized counseling services or a faxed referral to the quitline. Overall, 57 % accepted AR, 43 % accepted discharge medications, and 28 % accepted both. In one hospital, patients were less likely to accept the AR than another hospital, but more likely to accept medications and both counseling and medications (p < .001). Patients’ primary care providers were notified 76 % of the time, with nearly all providers notified in one hospital and less than half notified in another. About half of the intervention recipients completed call 1, and completion rates fell for each subsequent call.

### Massachusetts General Hospital (MGH)

Treatment *delivery* and *enactment* were both monitored by tracking acceptance and participation in treatment components among 529 intervention participants (see Table 7). In terms of treatment *delivery*, all 529 participants received one month of free medication at the time of hospital discharge, although only 51 % requested the 2nd month of medication (1st refill) and only 19 % requested the 3rd (2nd refill). All were enrolled into the TelASK IVR database and were called after hospital discharge and almost 9 out of 10 (88 %) accepted at least 1 IVR call. Success of transfer from the IVR call to counseling was also monitored. In the first two months of the study, 33 % of calls were dropped, in part due to participants hanging up prior to transfer completion. After adjustment that included reduced wait time and a recorded message to remain on the line, the IVR-to-counselor successful transfer rate increased and held steady reflecting a successful transfer in 83 % of patients requesting transfer to AWI. In terms of treatment *enactment*, about one-third of those who accepted at least one TelASK IVR call enrolled in telephone counseling through AWI, with 20 % completing all 5 outgoing counselor calls.

## Discussion

### *Study design* and *training*

There was little reported cross-contamination among the study arms. Quasi-experimental studies, such as the UMMC study can prevent intervention drift as they allow for greater separation of groups or treatment *differentiation*. UCSD and KU had fidelity measures of the usual care arm, which can measure treatment *differentiation* between arms. *Training* was universally applied across all of the studies and can be enhanced by developing manuals and/or toolkits for providers and patients as was done in the UMMC and NYU studies. *Training* enhances standardization across intervention activities [2] and reduces the risk of variation in strength and elements of intervention provided. *Training* increases competence, self-efficacy, and confidence [24, 25].

### Delivery/Receipt/ Enactment

Once *training* is complete, continued monitoring is necessary. Four of the studies (UMMC, KU, and NYU, KPCHR) used a checklist to monitor interventionists, but the items varied widely. The KU checklist (see Table 3) was largely based on specific components of their unique intervention. The UMMC checklist (see Appendix 1) was based on Joint Commission (JC) standards for inpatient smoking cessation interventions [26] and this approach can directly translate into quality assurance. The KPCHR checklist (see Table 6) was designed to capture key elements of the US Public Health Service [27] and JC standards for smoking cessation. The NYU checklist (see Appendix 2) was based largely on the relationship the counselor built with the patient, which is important as differences in warmth and ease, interactional style, and therapist empathy and self- efficacy can represent serious threats to fidelity [4, 28].

*Delivery* of medications ranged from 31 to 100 % across the studies, with higher levels coming from those studies that gave away free medications (UCSD and MGH) and lower levels coming from those studies that sought to obtain prescriptions for the patient in real world systems (NYU). *Delivery* of counseling was highest among those studies that used automated systems (websites and IVR systems) such as UAB, KPCHR, and MGH, but this did not automatically translate into treatment *receipt and enactment*. In the KU study, warm hand-off versus fax referral did increase enrollment in the quitline. In the UAB study most participants in the intervention arm accessed the website, but only about one-quarter participated in email communication.

Some studies measured treatment *enactment* in two ways showing concurrence or discordance between the two measures. In the NYU study, patient reports of participating in phone calls were only slightly lower than those reported by counselors. In the UAB study, the website reported fewer logins than a subset of patients self-reported, perhaps due to social desirability or recall bias, as patients were surveyed 6 months after discharge. Automated systems, such as the IVR, telephone, and website systems used in the KU, UAB, KPCHR, and MGH studies provide easily available fidelity data. Yet, automated data may not identify qualitative issues identified in observations of and surveys/interviews with interventionists and patients as done in the UMMC, KU, NYU, KPCHR, and other similar studies [29]. Unlike data from automated systems, qualitative data can provide rich information about barriers and facilitators to implementation.

In summary, all sites monitored their intervention and study fidelity, in particular how they differentiated between study arms, what training was provided and who engaged in it, what was delivered and to whom/how much, and to a lesser extent, intervention receipt and enactment by participants. Using these 5-categories as a guide, the studies all achieved monitoring of each aspect, to greater and lesser degrees as fit their programs. These examples may help other researchers in developing their own fidelity monitoring plans by identifying similar structural components and the methods for those components described herein.

### Challenges of fidelity monitoring

Recording treatment sessions and then having two observers rate fidelity is considered the gold standard [10], but can be very time consuming and costly, especially in dissemination trials [11]. Some might argue that fidelity monitoring fundamentally changes the intervention itself in that the fidelity assurance process serves as a reminder that would otherwise not exist. If the fidelity assessment must be viewed as part of the intervention, the validity of the intervention is challenged when it is conducted without fidelity checks once the research has been concluded and the intervention continues to be usual care.

Rigid application of treatment protocols may impede treatment delivery. The therapists may feel “locked in” or resistant to “cookbook” approach. Fidelity arguably does not allow the provider to tailor the intervention to the patient’s needs. For example, treatment fidelity may interfere with sound clinical judgment when shorter sessions or cultural adaptations to a treatment protocol are warranted. It may be a challenge to implement rigid protocols for smoking cessation in busy inpatient settings [3, 10, 30].

While modifying an intervention presents a challenge to fidelity measurement [11], a realistic and sustainable intervention may need to allow for adaptation and modification. Some studies make adaptability under certain circumstances a requirement [30–32]. While some researchers question that adaptations retain efficacy/effectiveness [2, 8], fidelity checks can be used to empower the stakeholders to actively participate in the process of adaptation. Finding the optimal balance between fidelity and adaptation requires a treatment design that allows for modifications and also calls for competence among interventionists to make appropriate treatment decisions [33].

## Conclusion

Despite the challenges of measuring fidelity, the seven CHART studies used a variety of methods to enhance fidelity with the goal of ensuring the accurate delivery of smoking interventions with consideration for feasibility and sustainability. The examples provided can be used to guide future studies. Some of the strategies to address fidelity may be more adaptable to certain types of trials and matching monitoring methods to study design and program deliverables will facilitate appropriate fidelity tracking. For example, direct observation may work best in RCTs where there are few interventionists, whereas direct observation is more difficult in large implementation trials that could instead use provider surveys. Researchers may determine which of the studies described is similar to their own and consider measuring fidelity in similar ways. The strengths, limitations, potential need for adaptation, and costs of fidelity monitoring need to be taken into consideration when designing and implementing clinical trials.

## Appendix 1

### (UMMC): Fidelity Nurse Interview Guide

**DURING HOSPITAL STAY:**

**Patient was asked about tobacco use.**

**Smoking status was documented.**

For patients, who are current tobacco users OR quit within the last 12 months:

**PATIENT WAS OFFERED COUNSELING DURING HOSPITAL STAY.**

Quit Smoking brochure was provided.

**PATIENT RECIEVED COUNSELING.**

Behavioral coments:

Advised the patient to set a quit date, ideally within 2 weeks

Advised the patient to remove all tobacco products from home and at work.

Discussed potential challenges to quitting, staying off tobacco, and planning ahead on how to deal with the challenges.

Provided the patient with strong messages of support and encouragement,

Stop Smoking videotape was provided.

Tobacco Tactics workbook was provided.

**Information about FDA-approved cessation medication was provided.**

**AT DISCHARGE:**

**Patient was offered referral to outpatient counseling.**

**Patient recieved referral to outpatient counseling.**

**Patient was offered prescription for FDA-approved cessation medication.**

**Patient received prescription for FDA-approved cessation medication.**

**Bolded items are Joint Commision standards.**

## Appendix 2

**Table 8:** (NYU): fidelity counseling audio review form

	Date:	Study ID#:		
	TTS:			
	Fidelity Rater:	Length of session: minutes		
	Counseling Session:			
	1 = 1st call 2 = Counseling 3 = Post-Quit			
DOMAINS	N/A	Did not do	OK	Did well
1. Treatment Alliance/Non-specific effects				
Outlines program for patients, obtains buy-in and forms working relationship.				
Demonstrates warmth. (E.g., “I can understand how you feel.”) Took a personal interest in pt.				
Demonstrates credibility. “E.g., “Many patients have found that distracting themselves by doing something pleasant can help them deal with the urge to smoke.”				
Minimizes non-relevant (e.g., non-smoking-related) conversation. (E.g., “I hear what you’re saying (reflect/summarize). Let’s shift and talk about your smoking.”				
Comments:				
2. Assessment				
Obtained history of smoking				
Environment: Assesses smoking at home and among friends and family				
Elicited pros and cons of smoking				
Elicited personal psychological/habit/physical components of addiction				
Discussed motivation to quit and helped patient expand on meaning of their reasons. “You mentioned health, what would be different if you had better health?” “What are some reasons that’s important for you?”				
Discussed confidence in ability to quit				
Comments:				
3. Counseling Skills				
Asks more open-ended questions. (E.g., “Let’s spend a few minutes talking about your quit date. Can you tell me about the plan for this day?”)				
Encourages change talk from patient (e.g., less talking by TTS and more listening). Rule of thumb: Patient should talk 3 times as much as the counselor.				
Asks one question at a time.				
Corrects assumptions and uses Elicit-Provide-Elicit framework (E.g., “May I tell you a little about the effects of smoking on ----?” “Actually, that’s what many people think, can I tell you more about that?”				
Uses empathic listening statements to Affirm patient’s experience (reflection: simple, complex as needed)				
Uses summaries/transition statements when introducing a new topic/idea. E.g., “I understand that you want to quit because of your health and for your children. These are very important reasons. Let’s switch to a different, but related topic and discuss some reasons why you *like* smoking.”				
Comments:				
4. Cessation Planning				
Elicited patient ideas and exchanges information about quit plan, including correcting assumptions.				
Discusses NRT – past use, type of use, corrected assumptions				
Helped patient set behavioral goals and a quit date				
Identified triggers for smoking (psychological/habit/physical) and environmental (family, friends who smoke)				
Helped patient identify strategies for coping with triggers				
Asks patient to review upcoming plan for the week in their own words/check in re understanding				
Gives encouragement. (E.g., “I know you can do this, and I’ll be here to help you.”)				
Reviews number of remaining sessions				
Problem-solving (follow up calls)				
Examines slip or relapse situations				
Discuss and normalize withdrawal symptoms				
Develops patient self-image as a non-smoker				
Comments:				
5. Adaptivity				
Tailors the content of counseling to *patient needs* (e.g., active/passive engagement style; information-seeking/avoidant; psychologically distressed/non-distressed; motivated/unmotivated.) E.g., Mental health/anxious patients may have difficulties with problem-solving and follow-through. These patients may need a more directive approach. Patients who are information-seeking may have lots of questions; TTS is able to answer these but can keep the counseling on course. Meets the patient where he/she is at.				
Tailors the content of counseling according to the *session flow* (e.g., fluidity, flexibility). If a patient has quit, and more time is spent talking about NRT, then spend a little less time on motivation building.				
Comments:				

## References

[1] Korda H (2013). Bringing evidence-based interventions to the field: the fidelity challenge. Journal of Public Health Management and Practice.

[2] Bellg AJ, Borrelli B, Resnick B, Hecht J, Minicucci DS, Ory M (2004). Enhancing treatment fidelity in health behavior change studies: Best practices and recommendations from the NIH behavior change consortium. Health Psychol.

[3] Stein KF, Sargent JT, Rafaels N (2007). Intervention research - Establishing fidelity of the independent variable in nursing clinical trials. Nurs Res.

[4] Campbell BK, Manuel JK, Manser ST, Peavy KM, Stelmokas J, McCarty D (2013). Assessing fidelity of treatment delivery in group and individual 12-step facilitation. J Subst Abus Treat.

[5] Guydish J, Campbell BK, Manuel JK, Delucchi KL, Le T, Peavy M (2014). Does treatment fidelity predict client outcomes in 12-Step Facilitationfor stimulant abuse?. Drug Alcohol Depend.

[6] Durlak JA, DuPre EP (2008). Implementation matters: a review of research on the influence of implementation on program outcomes and the factors affecting implementation. Am J Community Psychol.

[7] Santacroce SJ, Maccarelli LM, Grey M (2004). Intervention fidelity. Nurs Res.

[8] Elliott DS, Mihalic S (2004). Issues in disseminating and replicating effective prevention programs. Prev Sci.

[9] Borrelli B, Sepinwall D, Ernst D, Bellg AJ, Czajkowski S, Breger R (2005). A new tool to assess treatment fidelity and evaluation of treatment fidelity across 10 years of health behavior research. J Consult Clin Psychol.

[10] Borrelli B (2011). The assessment, monitoring, and enhancement of treatment fidelity in public health clinical trials. J Public Health Dent.

[11] McHugh RK, Murray HW, Barlow DH (2009). Balancing fidelity and adaptation in the dissemination of empirically-supported treatments: The promise of transdiagnostic interventions. Behav Res Ther.

[12] Spillane V, Byrne MC, Byrne M, Leathem CS, O’Malley M, Cupples ME (2007). Monitoring treatment fidelity in a randomized controlled trial of a complex intervention. J Adv Nurs.

[13] Moncher FJ, Prinz RJ (1991). Treatment fidelity in outcome studies. Clin Psychol Rev.

[14] Riley WT, Stevens VJ, Zhu S-H, Morgan G, Grossman D (2012). Overview of the consortium of hospitals advancing research on tobacco (chart). Trials.

[15] Duffy SA, Ronis DL, Titler MG, Blow FC, Jordan N, Thomas PL (2012). Dissemination of the nurse-administered Tobacco Tactics intervention versus usual care in six Trinity community hospitals: study protocol for a comparative effectiveness trial. Trials.

[16] Cummins S, Zhu S-H, Gamst A, Kirby C, Brandstein K, Klonoff-Cohen H (2012). Nicotine patches and quitline counseling to help hospitalized smokers stay quit: study protocol for a randomized controlled trial. Trials.

[17] Grossman E, Shelley D, Braithwaite RS, Lobach I, Goffin A, Rogers E (2012). Effectiveness of smoking-cessation interventions for urban hospital patients: study protocol for a randomized controlled trial. Trials.

[18] Harrington KF, McDougal JA, Pisu M, Zhang B, Sadasivam RS, Houston TK (2012). Web-based smoking cessation intervention that transitions from inpatient to outpatient: study protocol for a randomized controlled trial. Trials.

[19] Fellows JL, Mularski R, Waiwaiole L, Funkhouser K, Mitchell J, Arnold K (2012). Health and economic effects from linking bedside and outpatient tobacco cessation services for hospitalized smokers in two large hospitals: study protocol for a randomized controlled trial. Trials.

[20] Reid ZZ, Regan S, Kelley JHK, Streck JM, Ylioja T, Tindle HA (2015). Comparative effectiveness of post-discharge strategies for hospitalized smokers: study protocol for the Helping HAND 2 randomized controlled trial. BMC Public Health.

[21] Richter KP, Faseru B, Mussulman LM, Ellerbeck EF, Shireman TI, Hunt JJ (2012). Using “warm handoffs” to link hospitalized smokers with tobacco treatment after discharge: study protocol of a randomized controlled trial. Trials.

[22] Rigotti NA, Clair C, Munafo MR, Stead LF (2012). Interventions for smoking cessation in hospitalised patients. The Cochrane Database of Systematic Reviews.

[23] Rigotti NA, Regan S, Levy DE, Japuntich S, Chang Y, Park E (2014). Sustained care intervention and postdischarge smoking cessation among hospitalized adults: A randomized clinical trial. JAMA.

[24] Duffy SA, Karvonen-Gutierrez C, Ewing LA, Smith PM (2010). Veterans Integrated Services Network (VISN) 11 Tobacco Tactics Team. Implementation of the Tobacco Tactics Program in the Department of Veterans Affairs. J Gen Intern Med.

[25] Fore A, Karvonen-Gutierrez C, Talsma A, Duffy SA (2014). Nurses’ delivery of the Tobacco Tactics intervention at a Veterans Affairs Medical Center. J Clin Nurs.

[26] Joint Commission on Accreditation of Healthcare Organizations. Tobacco Treatment Measures (TTM). 2011; http://www.jointcommission.org/assets/1/6/Tobacco%20Treatment%20Measures%20List1.PDF. Accessed 2 February 2012.

[27] United States Public Health Service Commissioned Corps. Tobacco Smoking Cessation. 2011; http://ccmis.usphs.gov/ccbulletin/articles/smoking_cessation_02_2011.aspx. Accessed 23 March 2015.

[28] Campbell BK, Buti A, Fussell HE, Srikanth P, McCarty D, Guydish JR (2013). Therapist predictors of treatment delivery fidelity in a community-based trial of 12-step facilitation. Am J Drug Alcohol Abuse.

[29] Katz DA, Holman JE, Johnson SR, Hillis SL, Adams SL, Fu SS (2014). Implementing best evidence in smoking cessation treatment for hospitalized veterans: results from the VA-BEST Trial. The Joint Commission Journal on Quality and Patient Safety.

[30] Leventhal H, Friedman MA (2004). Does establishing fidelity of treatment help in understanding treatment efficacy? Comment on Bellg et al. (2004). Health Psychol.

[31] Addis ME, Krasnow AD (2000). A national survey of practicing psychologists’ attitudes toward psychotherapy treatment manuals. J Consult Clin Psychol.

[32] Barlow DH, Levitt JT, Bufka LF (1999). The dissemination of empirically supported treatments: a view to the future. Behav Res Ther.

[33] Backer T (2001). Finding the balance: program fidelity and adaptation in substance abuse prevention: A state-of-the-art review.

